# Quantitative and Qualitative Pain Evaluation in Response to OnabotulinumtoxinA for Chronic Migraine: An Observational Real-Life Study

**DOI:** 10.3390/toxins15040284

**Published:** 2023-04-15

**Authors:** Claudia Altamura, Nicoletta Brunelli, Giovanna Viticchi, Sergio Salvemini, Gianluca Cecchi, Marilena Marcosano, Luisa Fofi, Mauro Silvestrini, Fabrizio Vernieri

**Affiliations:** 1 Unit of Headache and Neurosonology, Department of Medicine and Surgery, Università Campus Bio-Medico di Roma, Via Alvaro del Portillo, 21, 00128 Roma, Italy; c.altamura@policlinicocampus.it (C.A.);; 2 Fondazione Policlinico Universitario Campus Bio-Medico, Via Alvaro del Portillo, 200, 00128 Roma, Italy; 3 Neurological Clinic, Marche Polytechnic University, Via Conca 1, 60020 Ancona, Italy

**Keywords:** OnabotulinumtoxinA, chronic migraine, prevention, pain intensity, pain quality

## Abstract

(1) Background: Randomized controlled trials and real-life studies demonstrated the efficacy of OnabotulinumtoxinA (OBT-A) for CM prevention. However, no studies specifically addressed its effect on pain’s quantitative intensity and qualitative characteristics. (2) Methods: This is an ambispective study: a post-hoc retrospective analysis of real-life prospectively collected data from two Italian headache centers on CM patients treated with OBT-A over one year (i.e., Cy1-4). The primary endpoint was the changes in pain intensity (Numeric Rating Scale, NRS; the Present Pain Intensity (PPI) scale, the 6-point Behavioral Rating Scale (BRS-6)) and quality scale (the short-form McGill Pain Questionnaire (SF-MPQ)) scores. We also assessed the relationship between changes in intensity and quality of pain and disability scale (MIDAS; HIT-6) scores, monthly headache days (MHDs), and monthly acute medication intake (MAMI) (3) Results: We retrieved 152 cases (51.5 years SD 11.3, 80.3% females). From baseline to Cy-4, MHDs, MAMI, NRS, PPI, and BRS-6 scores decreased (consistently *p* < 0.001). Only the throbbing (*p* = 0.004), splitting (*p* = 0.018), and sickening (*p* = 0.017) qualities of pain collected in the SF-MPQ were reduced. Score variations in MIDAS related to those in PPI scales (*p* = 0.035), in the BRS-6 (*p* = 0.001), and in the NRS (*p* = 0.003). Similarly, HIT-6 score changes related to PPI score modifications (*p* = 0.027), in BRS-6 (*p* = 0.001) and NRS (*p* = 0.006). Conversely, MAMI variation was not associated with qualitative or quantitative pain score modifications except BRS-6 (*p* = 0.018). (4) Conclusions: Our study shows that OBT-A alleviates migraine by reducing its impact on multiple aspects, such as frequency, disability, and pain intensity. The beneficial effect on pain intensity seems specific to pain characteristics related to C-fiber transmission and is associated with a reduction in migraine-related disability.

## 1. Introduction

Migraine is a neurological condition leading to significant disability despite its paroxysmal pattern and benign course [1]. Migraine can be diagnosed upon defined clinical criteria [2]. It occurs as recurrent headache attacks of pulsating pain of moderate to severe intensity, which is aggravated with movement and is associated with bothersome accompanying symptoms such as nausea and photo- and phonophobia. During a lifetime, migraine attacks can present with a fluctuating frequency influenced by several modifying and non-modifying factors that are not entirely clarified [3]. Some patients (around 8%) experience a progressive increase in attack frequency until they occur 15 days per month or more [4]. Several factors are hypothesized to promote the progression to a chronic condition [5], including frequent acute medication intake [6].

On the other hand, patients with CM can spontaneously revert to an episodic frequency [7]. People with chronic migraine (CM, ICHD-3) [8] endure a severe disability as pain is experienced as part of a constellation of disturbances, including other non-cranial pain, emotional distress, sleep, gastrointestinal, and other somatic disorders [9,10]. The frequent occurrence of migraine attacks produces the release of molecules with neuroinflammatory properties and neurotransmitters outside the blood–brain barrier (BBB), which enhance the activation of first-order trigeminal nociceptive neurons (i.e., peripheral sensitization) [11]. Moreover, especially when associated with comorbid conditions, it is believed to lead to maladaptive changes and impaired trigeminovascular functions, which comprise the “central sensitization” processes, resulting in modifications in subjective pain perception [12]. Central sensitization is the abnormal responsiveness of central nociceptive neurons to subthreshold afferent inputs, a phenomenon primarily theorized in migraine [13]. As a consequence, patients with CM can often experience headaches that are not characterized by typical pulsating migraine features [8] and may develop allodynia [14]. The throbbing pain is likely due to the release of vasoactive substances during migraine attacks, also mediated by mechanotransducers (e.g., Piezo channels [15]), while the origin of the non-throbbing headache, which is often reported in CM patients, is not entirely understood.

Randomized controlled trials and real-life studies have demonstrated the efficacy, safety, and tolerability of OnabotulinumtoxinA (OBT-A), when injected according to the PREEMPT protocol, in CM prevention [16,17,18,19,20]. The exact mechanisms subtending the relief from a headache are not entirely clarified. However, different pathways probably mediate the OBT-A beneficial effect [21,22]. First, OBT-A inhibits the exocytosis of vehicles releasing pro-inflammatory and excitatory neurotransmitters and neuropeptides such as substance P, calcitonin-gene-related peptide (CGRP), and glutamate from primary afferent sensory C fibers transmitting nociceptive pain during migraine attacks and contributing to the development of peripheral sensitization. OnabotulinumtoxinA also decreases the insertion of pain-sensitive ion channels into the synaptic membranes of nociceptive neurons, resulting in reduced sensory neuron excitability. This inhibition of peripheral sensory fiber activation antidromically decreases the rate of pain signaling directed from the dura to the spinal trigeminal nucleus, which in turn hinders the establishment of hyperexcitability (i.e., central sensitization) of the neural networks involved in migraine pathophysiology [23]. This secondary effect on central sensitization is clinically evident in the progressive benefit observed in treated patients [16]. It is also supported by the finding that allodynia represents a favorable prognostic factor for a good outcome after OBT-A [24].

Previous studies demonstrated that OBT-A decreased headache days and pain intensity [25], but no studies have specifically investigated its effect on the qualitative characteristics of headache pain in treated patients. The experience of pain goes far beyond mere nociception; it also encompasses affective, cognitive, and judgmental processes [26]. The recurrence of moderate or severe pain can provoke anticipatory fear and avoidance behavior, contributing to migraine-associated disability [27].

This study investigated the quantitative and qualitative changes in pain perception in CM patients receiving one-year treatment with OBT-A.

## 2. Results

For the present analysis, we retrieved the data of 152 CM patients (51.5 years SD 11.3, 122 females, 80.3%) with a complete data set from a pool of clinical dossiers of 236 patients. All included patients had received at least three previous preventive therapies and reported an inadequate response. Of these, 19 patients were excluded because of incomplete data regarding primary variables, as well as 65 cases for not completing a one-year OBT-A treatment yet. All patients were treated with Botox^®^. Table 1 summarizes the variation in clinical variables and pain scales from baseline to Cy-4. As shown, patients achieved a significant decrease in monthly headache days (MHDs, by 7 days), monthly acute medication intake (MAMI, by 6 drugs), and pain intensity scales. In particular, Numeric Rating Scale (NRS) scores were reduced by 0.9 points out of 10, the Present Pain Intensity (PPI) scale scores by 0.3 out of 5 points, and the Behavioral Rating Scale (BRS-6) scores by 0.5 grades out of 6. These variations were consistently significant (*p* < 0.001). On the other hand, there was a reduction only in the throbbing (by 0.3 points; *p* = 0.004), splitting (by 0.3 points; *p* = 0.018), and sickening (by 0.3 points; *p* = 0.017) qualities of pain according to the McGill Pain Questionnaire (SF-MPQ).

**Figure 1:**
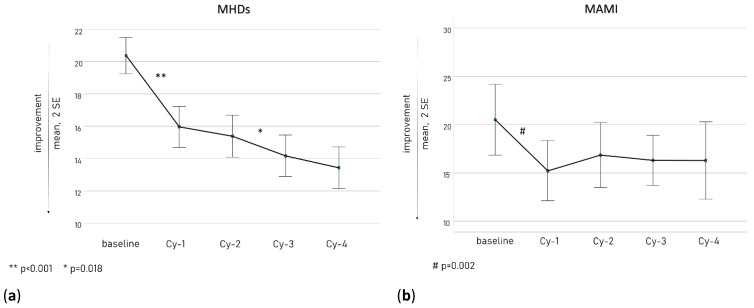
The MHD (**a**) and MAMI (**b**) course along the treatment cycles.

Figure 1 shows the course of MHDs (a, left panel) and MAMI (b, right panel) along the treatment cycles. As illustrated, the principal reduction in MHDs and MAMI took place at Cy-1 (*p* < 0.001 and *p* = 0.002, respectively), even though MHDs presented a further decrease along the following treatment cycles (from Cy-2 to Cy-3 *p* = 0.018).

**Figure 2:**
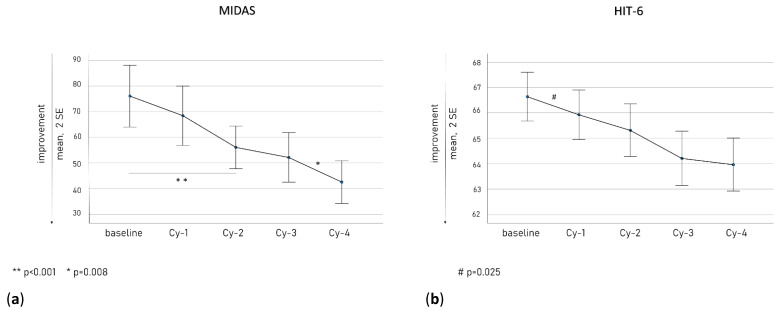
The MIDAS (**a**) and HIT-6 (**b**) scores along the treatment cycles.

Figure 2 illustrates the MIDAS (a, left panel) and HIT-6 (b, right panel) scores along the treatment cycles. Their decline was gradually constant, with a significant reduction in HIT-6 scores at Cy-1 (*p* = 0.025) and in MIDAS scores at Cy-2 (*p* = 0.001) compared to baseline.

**Figure 3:**
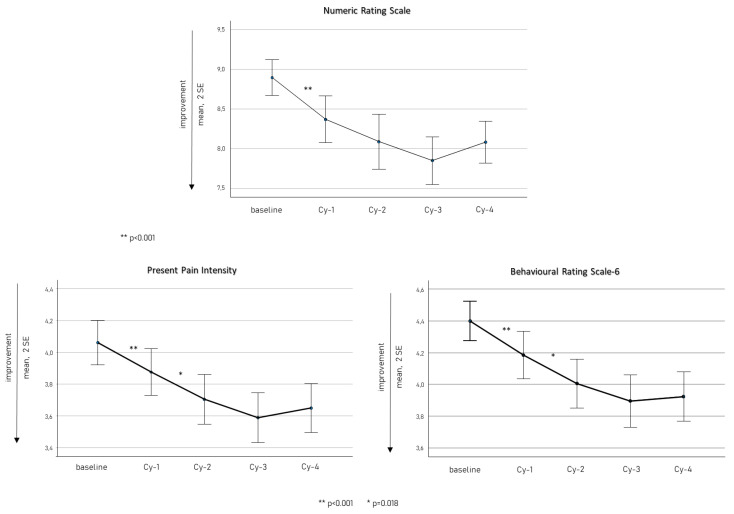
Quantitative pain scale scores (NRS, PPI, and BRS) from baseline to Cy-4.

Figure 3 displays quantitative pain scale scores (NRS, PPI, and BRS) from baseline to Cy-4. They presented a significant reduction within Cy-1 (consistently *p* < 0.001), and PPI and BRS showed a further significant decrease from Cy-1 to Cy-2 (both *p* = 0.018).

**Figure 4:**
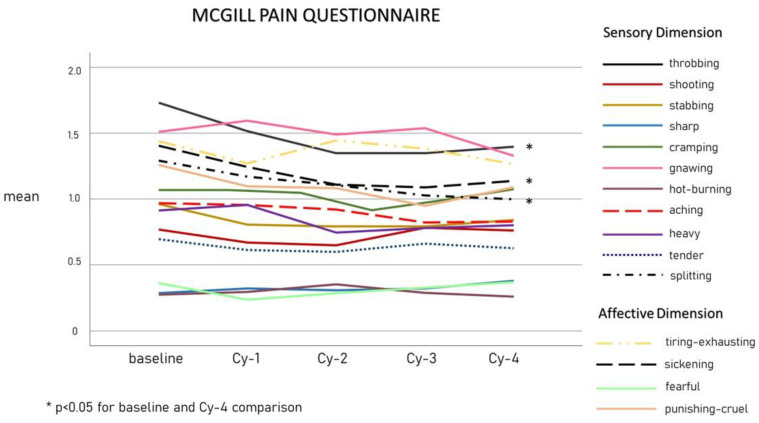
The means of SF-MPQ qualitative pain scores from baseline to Cy-4.

Figure 4 shows the trends of SF-MPQ qualitative pain scores from baseline to Cy-4. The intensity of throbbing and sickening pain decreased within Cy-1 (*p* = 0.005 and *p* = 0.044, respectively), while the reduction in splitting pain intensity became significant at Cy-4 (*p* = 0.018).

The intensity of the different pain qualities as described at baseline did not differ in patients achieving MHD 30% RR or MHD 50% RR at Cy1- and Cy-4 compared to baseline (*p* > 0.05 consistently for all comparisons).

We found a significant correlation between the changes from baseline to Cy-4 in disability questionnaire scores and quantitative intensity pain scores. In particular, score variations in MIDAS related to those in PPI scales (*p* = 0.035, Pearson’s correlation = 0.184), in BRS-6 (*p* = 0.001, Pearson’s correlation = 0.276), and in NRS (*p* = 0.003, Pearson’s correlation = 0.256). Similarly, HIT-6 score changes related to PPI score modifications (*p* = 0.027, Pearson’s correlation = 0.183), in BRS-6 (*p* = 0.001, Pearson’s correlation = 0.283), and NRS (*p* = 0.006, Pearson’s correlation = 0.229). Conversely, we did not observe any correlation between the changes in qualitative pain and disability scale scores. In addition, MAMI was not associated with qualitative or quantitative pain score modifications except for BRS-6 (*p* = 0.018, Pearson’s correlation = 0.197).

## 3. Discussion

The introduction of OBT-A in the treatment of chronic migraine after the encouraging results of the PREEMPT studies [28,29,30] was the first strategy offering an effective therapy without significant side effects [31]. Real-life studies confirmed these benefits in more than ten-year clinical experiences [32,33,34].

The results observed in the present one-year observational study further demonstrate the beneficial impact of OBT-A on all the clinical parameters evaluated: monthly days of headache, acute medication intake, pain intensity, and migraine-related disability. The benefit was mainly observed after the first cycle but further increased in magnitude during the treatment year. Moreover, even patients who did not achieve MHD 30% or 50% RR reported a significant benefit for pain intensity, presenting mild, not disabling, attacks. This aspect is often neglected when considering the outcome of migraine-preventive treatments [35].

The therapies specifically targeting CGRP opened more opportunities in migraine management [36] with rapid and consistent improvement in patients with a long disease history and previous treatment failures, as confirmed by real-life studies [37,38,39]. However, even with this highly specific and effective approach, up to 40% of patients do not report a 50% reduction in MHDs, claiming more therapeutic strategies [40]. 

An increasing number of reports propose for hard-to-treat patients a combined strategy of OBT-A and monoclonal antibodies targeting the CGRP pathway [41,42,43] based on the fact that they play synergistic effects within the trigeminovascular system, with monoclonal antibodies inhibiting the activation of Aδ fibers and OBT-A that of C fibers [44]. These two types of fibers have a key role in pain perception along the trigeminal pathway. In the trigeminal ganglion, up to 50% are CGRP-positive (releasing) C fibers, while around 1/3 display CGRP receptors (Aδ fibers) [36]. Their coexistence in the trigeminal ganglion is a fundamental mechanism in pain amplification. The myelinization degree of C (unmyelinated, slow-conducting) and Aδ (lightly myelinated, more rapidly conducting) fibers explain the different qualities of pain transmission. While C fibers are considered to transmit aching, throbbing, or burning pain that build up slowly, the Aδ fibers conduct initial, sharper pain [45]. In this scenario, evaluating the effect of OBT-A on pain quality would enhance knowledge on its mechanism of action. 

In line with the hypothesized OBT-A inhibition on CGRP-releasing C fibers, the patients in our cohort reported a more rapid and higher reduction magnitude in throbbing pain quality, which in turn also had the highest intensity scores at baseline (Table 1, Figure 4). This observation is certainly consistent with the migraine diagnosis itself, as pulsating/throbbing pain is one of the main diagnostic criteria [8]. Patients also reported, after the first cycle, an improvement in the sickening pain quality. Although recorded among the affective dimension of pain, it is possible that patients associated the adjective “sickening” with the disturbing nausea they perceive during the attacks, which is possibly related to CGRP neuron activation in the area postrema [46]. Finally, we observed a decrease in the “splitting” quality of pain intensity, even though this became significant only in the last treatment cycle. The splitting pain quality was described as “an intense sensation of discomfort or distress that feels like being cut apart,” which may resemble unilateral migraine pain. It should be noted that unilateral pain represents a favorable prognostic factor for OBT-A [24]. 

The effect exerted by OBT-A on throbbing, sickening, and splitting pain qualities seems to reflect with high specificity the mechanisms subtending the trigeminal vascular activation during migraine attacks. Few studies have investigated pain characteristics in patients treated with OBT-A. Imploding pain (described as a sensation of being crushed, clamped, or stubbed by external forces) was more often reported in responders to OBT-A than non-responders [47]. In our cohort, responders to Cy-1 or Ct-4 did not present different pain quality compared to non-responders. However, our patients were not specifically required to describe the pain as imploding compared to exploding, so we could not confirm or confute the predictive value of such pain qualities. Moreover, our real-life cohort consisted of patients who perceived a clinical benefit, which allowed us to confirm the clinical indication for OBT-A for the treatment year.

In our cohort, the primary therapeutic advantage was achieved mainly within Cy-1 as observed in terms of reduction in MHDs and MAMI (Figure 1), disability scale scores (Figure 2), and quantitative pain evaluation (Figure 3). A short-term reduction in pain intensity was described as a positive predictive factor of response in the long term [48,49]. It is possible that an initial decrease in pain intensity is the most sensitive marker of OBT-A efficacy, in accordance with the more rapid inhibition achieved at the peripheral C-fiber level. This aspect should arouse particular interest. In the perspective of a tailored therapeutic approach, in light of the new CGRP targeted strategies, early markers of OBT-A efficacy should be carefully investigated in treated patients to avoid unnecessarily prolonged treatment regimens. Therefore, a detailed quantitative and qualitative assessment of pain after the first OBT-A cycle would allow a more comprehensive and rapid evaluation of its efficacy and should be implemented when prescribing other treatment regimens. Nevertheless, we also observed a progressive increase in OBT-A’s beneficial effects, possibly as a consequence of gradual central de-sensitization, allowing an increase in the pain threshold [23,50]. 

Interestingly, we found a correlation between the reduction in pain intensities and disability scales, suggesting that both short- and long-term pain alleviation may have a relevant impact on the perceived migraine burden. Again, these results underline the importance of fully including pain assessment beyond the mere MHD reduction in defining the response to migraine preventive treatment, especially in patients with CM [16,35]. This point of view could also provide actionable information for patients. The emotional aspects of pain play a key role in pain perception. Around 25% of migraine patients feel anxiety and negative emotions accompanying or preceding attacks; this affective phenomenon is defined as “pain catastrophizing” [51]. In our cohort, the decrease in pain intensity was not related to a reduction in MAMI. This aspect deserves further consideration. On the one hand, it reflects the well-known phenomenon of presenteeism in migraine [52], where patients are used to overtreating pain in an attempt to keep up their productive activities. It may also be related to the psychopathological aspects that characterize CM patients with medication overuse [52]. Pain is not only a sensitive phenomenon but involves reward and motivational circuitry, where the pain is interpreted as punishment, and its relief generates a negative reward [26,53]. In this context, having patients become aware of improving pain scores after treatment can have positive reinforcement, in line with the encouraging findings obtained by adding acceptance and commitment therapy to the standard of care [54]. Interestingly, in patients treated with OBT-A, a reduction in pain catastrophizing significantly predicted a decline in headache frequency [55].

The main limitation of the study is that we only enrolled patients who successfully completed the one-year treatment; this did not allow us to correctly investigate if the qualitative and quantitative pain evaluation has a prognostic value in the outcome of OBT-A therapy. Another relevant limitation of our study is that we have not specifically addressed allodynia at baseline and during the treatment year. The impact of OBT-A on allodynia would have provided a greater insight into the central effect of the therapy. Similarly, when evaluating the pain intensity, patients were required to score the worst intensity but not its persistence during the attacks, giving a partial view of the whole burden of migraine [56].

## 4. Conclusions

Our study shows that OBT-A alleviates migraine by reducing its impact on multiple aspects, such as frequency, disability, and pain intensity, within the first cycle of therapy. The beneficial effect on pain intensity seems specific to pain characteristics related to C-fiber transmission and is associated with a reduction in migraine-related disability.

## 5. Materials and Methods

This is a post hoc ambispective study: a retrospective analysis of patient-level real-life data prospectively collected for clinical evaluation from two headache centers in Rome and Ancona. The inclusion criteria for the present analysis were (a) CM diagnosis according to the International Classification of Headache Disorders criteria [8]; (b) age 18 years old or older; (c) treatment with OBT-A 155–195 units quarterly according to the Phase 3 Research Evaluating Migraine Prophylaxis Therapy (PREEMPT) protocol for at least four cycles [28,57,58]. Exclusion criteria: (a) other chronic pain syndromes treated with analgesics for at least 15 days per month; (b) use of recreational drugs.

### 5.1. Data Collection

Medical records were reviewed to collect demographics and medical data, including migraine-related data. For standard clinical practices, all patients at our headache centers are well-instructed to prospectively fill in headache diaries to collect monthly headache days (MHDs), acute medication intake (MAMI) and disability questionnaires (Headache Impact Test, HIT-6 [59], monthly, and the Migraine Disability Assessing Scale, MIDAS [60], quarterly). In addition, to gain a better comprehension of the clinical response to OBT-A, patients fill out other clinical scales for pain evaluation: the Pain Numeric Rating Scale (NRS) for the most painful attack in a given month, the Present Pain Intensity (PPI) scale, the 6-point Behavioral Rating Scale (BRS-6) [61], and the short-form McGill Pain Questionnaire (SF-MPQ) [62,63].

The Numerical Rating Scale (NRS) is an 11-point scale for self-reported pain. It is the most commonly used unidimensional pain scale. The respondent selects a whole number (integers 0–10) that best reflects the intensity of his/her pain. The anchors are 0 = no pain and 10 = extreme pain/worst possible pain [64,65,66].

The PPI score is recorded as a number from 1 to 5, in which each number is associated with the following words: 1. Mild, 2. Discomforting, 3. Distressing, 4. Horrible, and 5. Excruciating. Both the NRS and the PPII provide data on pain intensity only and no data on the qualities of the pain [67].

The BRS-6 scale describes the limitations of activities because of pain, and it is articulated in 6 grades: 1. No pain, 2. Pain is present but does not limit my activities, 3. Can do most activities, 4. Unable to do some activities, 5. Unable to do most activities, and 6. Unable to do any activities.

The main component of the SF-MPQ consists of 15 pain quality descriptors (11 sensory: throbbing, shooting, stabbing, sharp, cramping, gnawing, hot-burning, aching, heavy, tender, splitting, and 4 affective: tiring—exhausting, sickening, fearful, and cruel—punishing), which are rated on an intensity scale as 0 = none, 1 = mild, 2 = moderate, or 3 = severe. The pain quality descriptors in the short-form McGill Pain Questionnaire were translated into Italian by a native speaker of Italian and English. A short description accompanied each adjective following MedGen, NCBI’s portal to information about conditions and phenotypes (www.ncbi.nlm.nih.gov/medgen/, accessed on 12 March 2023).

The MMD and MAMI baselines were defined as the monthly means of the 3 months preceding the first cycle of OBT-A treatment (Cy-1). The collected disability scales and questionnaires on pain intensity and quality filled out on the day of the first treatment cycle were considered baseline. All variables were collected at baseline, quarterly at each treatment cycle, and three months after the last cycle (Cy 1-4).

The primary endpoint was the observation of changes in pain intensity and quality scale scores during one year of OBT-A treatment. The secondary endpoints included assessing the relationship between changes in intensity and quality of pain and changes in disability scale scores, in MHDs, expressed as the percentual variation from baseline to Cy-4, and in MAMI intake, expressed as the absolute variation from baseline to Cy-4.

We considered as responders patients achieving at least a 30% reduction in MHDs (MHD 30% RR) in accordance with the Guidelines for CM trial [68] and as high responders those achieving at least a 50% reduction in MHDs (MHD 50% RR). We had defined such cut-offs in a population with at least three treatment insufficient responses; even a reduction of 30% in headache frequency has a clinically significant impact, especially if accompanied by relevant pain relief.

Ethical committees of the 2 participating centers were informed about our retrospective study, ad hoc data collection, and analysis. Patients signed informed consent to treatment and data management for the research aim [16]. The study was performed in accordance with the Helsinki Declaration of 1964 and its later amendments.

### 5.2. Statistical Analysis

This was a post hoc analysis. No sample size calculation was performed, as the sample size was based on the available data from a convenience sample. However, to achieve a power of 80% and a significance level of 5% (two-sided), for detecting an effect size of 0.3 between paired variables, a sample size of at least 112 subjects would have been necessary. An effect size of 0.3 reflects the cut-off of a 30% reduction in MHDs as clinically meaningful [67]. Statistical analyses were performed with SPSS version 27.0 (SPSS Inc., Chicago, IL, USA). The interval variables were expressed as means with standard deviations [SD]). A paired t-test was adopted to analyze the variable changes over time. The relationship between changes in intensity and quality pain clinical and disability scale scores were assessed using Pearson correlation.

Logistic regression (forced entry) was used to investigate if the intensity and quality of pain scores were related to disability scores independently of MHDs. We included only subjects with complete information regarding the primary studied variables. All tests were two-tailed. Statistical significance was set as *p* < 0.05.

## Figures and Tables

**Table 1:** Longitudinal changes in clinical variables and pain scales from baseline to Cy-4.

Mean (SD)	Baseline	Cy-4	*p*
Female *n*, (%)	122 (80.3)		
Age	51.5 (11.3)		
Monthly Headache Days	20.4 (6.9)	13.4 (7.8)	**<0.001**
Monthly Acute Medication Intake	20.9 (14.3)	14.3 (14.4)	**<0.001**
MIDAS	74.1 (64.7)	42.6 (44.9)	**<0.001**
HIT-6	66.7 (5.7)	64.0 (6.2)	**<0.001**
NRS	8.9 (1.4)	8.07 (1.6)	**<0.001**
PPI	4.0 (0.8)	3.7 (0.9)	**<0.001**
BRS-6	4.4 (0.7)	3.9 (0.9)	**<0.001**
**mean (SD)**	**baseline**	**Cy-4**	***p***
**McGill Pain Questionnaire**			
*Sensory dimension of pain experience*			
Throbbing	1.7 (1.3)	1.4 (1.2)	**0.004**
Shooting	0.8 (1.2)	0.8 (1.2)	0.956
Stabbing	0.9 (1.2)	0.8 (1.2)	0.444
Sharp	0.3 (0.8)	0.4 (0.9)	0.313
Cramping	1.0 (1.3)	1.1 (1.2)	0.797
Gnawing	1.5 (1.4)	1.3 (1.9)	0.389
Hot-burning	0.3 (0.8)	0.3 (0.7)	1.000
Aching	0.9 (1.3)	0.8 (1.2)	0.242
Heavy	0.9 (1.3)	0.8 (1.2)	0.281
Tender	0.7 (1.1)	0.6 (1.0)	0.484
Splitting	1.3 (1.4)	1.0 (1.3)	**0.018**
*Affective dimension of pain experience*			
Tiring—exhausting	1.3 (1.4)	1.2 (1.3)	0.254
Sickening	1.4 (1.3)	1.1 (1.2)	**0.017**
Fearful	0.4 (0.9)	0.4 (0.9)	0.748
Punishing—cruel	1.4 (1.4)	1.3 (1.3)	0.218

Highlighted in bold are values for which *p* < 0.05.

## Data Availability

Anonymized data will be shared upon request from any qualified investigator.
